# Mycorrhizal control of microbial gene transcription and taxonomic composition in the rhizosphere and bulk soil

**DOI:** 10.1093/ismejo/wraf282

**Published:** 2025-12-19

**Authors:** Fergus Wright, Stéphanie Grand, Ian Sanders, Ricardo Arraiano-Castilho

**Affiliations:** Department of Ecology and Evolution, University of Lausanne, Lausanne 1015, Vaud, Switzerland; Institute of Earth Surface Dynamics, University of Lausanne, 1015, Vaud, Switzerland; Department of Ecology and Evolution, University of Lausanne, Lausanne 1015, Vaud, Switzerland; Department of Ecology and Evolution, University of Lausanne, Lausanne 1015, Vaud, Switzerland

**Keywords:** soil microbiome, microbial interactions, AMF, bacteria, *zea mays*, *rhizophagus irregularis*, metatranscriptomics

## Abstract

Interactions between arbuscular mycorrhizal fungi (AMF) and soil microbial communities that support plant nutrient acquisition remain poorly understood. Here, we investigate how the model AMF species *Rhizophagus irregularis* influences microbial mRNA transcription and microbial taxonomic composition in rhizosphere and bulk soil compartments of *Zea mays* mesocosms. Using metatranscriptomic profiling alongside 16S rRNA and ITS amplicon sequencing, we show that AMF alter bacterial gene expression without shifting community composition and significantly increase fungal richness and evenness. We identify genotype-specific effects of AMF on microbial diversity and function and find that AMF colonisation stimulates microbial B-vitamin biosynthesis. We also link elevated plant leaf phosphorus levels under AMF colonisation with changes in root gene expression and increased abundance of AMF-stimulated rhizosphere bacterial taxa. These findings highlight the importance of feedback loops between plant, AMF and soil microorganisms and show how these interactions can contribute to increases in plant nutrient uptake. It is hoped these results will be useful for sustainable crop production and ecosystem regeneration through microbiome-informed management strategies.

## Introduction

The relationships between plant roots and the soil microbiome play an important role in global nutrient cycling, plant health, and ecosystem stability [1]. A deeper understanding of these interactions is urgently needed to advance sustainable agricultural practices and restoration ecology in the face of anthropogenic damage. Arbuscular mycorrhizal fungi (AMF) are a group of obligate symbionts, ubiquitous in terrestrial ecosystems, that form mutualistic associations with the roots of over 70% of all known plant species [2]. These fungi provide plants with essential nutrients and water in exchange for an estimated 4%–20% of photosynthesised plant carbon [3]. Their hyphae extend out from the rhizosphere (i.e. soil in the direct vicinity of plant roots) into the bulk soil (i.e. the soil compartment inaccessible to plant roots). In the rhizosphere, plant root exudates support a diverse community of microbes [4]. Whereas the bulk soil, without a direct supply of plant carbon, has lower rates of microbial activity and nutrient turnover [5], there is evidence to suggest that bulk soil microbial activity is enhanced in the presence of AMF hyphae [6–8]. This zone of interaction surrounding AMF hyphae and spores, referred to as the mycorrhizosphere, is increasingly recognised as a distinct ecological niche. It harbours mycorrhizal helper bacteria, plant growth promoting bacteria, facilitates microbial mobility along fungal hyphae, and receives organic inputs through exudates and decaying hyphal material [9, 10]. Specific microbial communities are often found strictly associated with AMF mycelia, and recently it has been shown bacteria from these communities can colonise the roots as endophytes [11, 12], suggesting that the mycorrhizosphere may function as a dynamic interface linking the soil microbiome to the rhizosphere. Despite these insights, our understanding of how such biotic interactions shape microbial communities, nutrient mineralisation, and plant growth remains limited.

It is well known that different AMF genotypes of the same species differ in their effects on plant growth, plant gene regulation and other traits such as extraradical hyphal production [13–15] that would likely result in differing interactions with the soil microbiome. One driver of AMF-microbiome interactions are hyphal exudates which have been shown to differ between AMF genotypes in response to nutrient concentrations in the immediate vicinity of hyphae [16]. Exudates could activate given components of the microbial community resulting in enhancement of certain functions. For instance, bacterial lytic enzymes able to degrade organic material and mineralise nutrients for hyphal uptake, have been shown to be enhanced in the presence of AMF in vitro [17, 18]. Previous studies have found enrichment of certain bacterial taxa associated with AMF hyphae. For instance, maize plants (*Zea mays* L.) inoculated with AMF and pulse labelled with ^13^CO_2_, showed substantial enrichment of ^13^C in bulk soil-inhabiting bacterial taxa thought to be specific to AMF stimulation [19]. Furthermore, using 16S rRNA sequencing, it has been shown that distinct bacterial communities associate with AMF and that this is consistent across soil types [20] and specific to AMF genotypes [21]. In separate studies, bacteria have been shown to form biofilms on hyphae [22] and migrate along the water-film surrounding hyphae [6], contributing to nutrient mineralisation [6, 17, 18]. Moreover, the role of rhizosphere inhabiting saprotrophic fungi may also have a significant effect on the turnover of organic matter in the rhizosphere, with evidence to suggest these fungi can recycle immobilised carbon and nutrients from dead AMF hyphae [23].

If AMF directly affect the taxonomic composition in the rhizosphere and bulk soil, this would in turn influence the composition of genes expressed by the microbiome in each compartment, since distinct microbial taxa typically harbour distinct sets of metabolic genes. In cases where taxonomic shifts lead to parallel changes in gene expression, the taxa associated with AMF would likely be performing functions directly linked to maintenance of the symbiosis. Alternatively, AMF could indirectly alter the taxonomic composition of microbial communities through changes to the soil environment that drive differences in transcriptional activity or community structure. Even if AMF do not change the taxonomic composition, they may still influence microbial function. This is possible because many metabolic traits are broadly distributed across phylogenetically diverse taxa, such that different subsets of the community can maintain similar functional outputs. This phenomenon, known as functional redundancy [24], would be reflected in cases where community composition remains stable, but transcriptional profiles differ. In both scenarios, shifts in transcribed genes would indicate changes in the metabolic capabilities of the microbiome with implications for soil biogeochemical processes.

Understanding how AMF affect soil bacterial and fungal mRNA transcription and taxonomic composition in the rhizosphere and bulk soil will be crucial to untangling the soil biogeochemical processes which lead to the known benefits of AMF e.g. increases in soil aggregation, organic carbon content and water retention [25–27]. Assessing interactions at the community level better captures the cumulative effect of AMF mediated microscale interactions that likely lead to these known benefits. A better understanding of cross-kingdom interactions would allow for management of agricultural and restoration ecosystems with the aim of synchronising community microscale interactions to maximise aboveground productivity with minimal inputs, mimicking natural ecosystems.

To develop our understanding of plant-AMF-soil microbial interactions at the community level, we sought to distinguish the effect of two AMF genotypes on the microbiome in the rhizosphere and the bulk soil compartments in the context of nutrient uptake in *Z. mays* and assess how soil microbial community transcribed mRNAs and taxonomic composition in the two soil compartments supports the mycorrhizal symbiosis and maize growth. To achieve this, we inoculated *Z. mays* plants with two different genotypes of *Rhizophagus irregularis* in controlled conditions and allowed a stochastically assembled microbial community from the background environment to establish. We profiled the AMF associated soil microbial transcribed mRNAs and taxonomic composition using targeted ribosomal RNA gene amplicon sequencing and metatranscriptome sequencing of mRNA from the different soil compartments. We hypothesised that AMF restructure plant–soil-microbiome interactions by shaping microbial community assembly and function in ways that enhance plant nutrient acquisition to maintain a functioning symbiosis. Specifically, (i) because soil compartments (rhizosphere and bulk soil) represent distinct ecological niches, AMF presence and genotype selectively influence microbial community composition across compartments, leading to compartment-specific taxa associations (ii) AMF will alter microbial functional gene expression, particularly in pathways related to nutrient mobilisation and rhizosphere metabolic activity, reflecting shifts in soil ecosystem functioning; (iii) Enhanced pant phosphorous uptake under AMF inoculation is mediated by coordinated changes in pant transcription and microbial community structure, linking symbiotic efficiency with microbiome-driven nutrient cycling.

## Materials and methods

### Experimental design

We inoculated 45 *Z. mays* (B73) plantlets (initially grown in vitro from surface sterilised seeds) with a suspension of 500 spores of one of two genotypes of the AMF species *Rhizophagus irregularis* (DAOM197198 (herein DAOM) and C2) or with water as a noninoculated control (CTL) 7 days after they had been planted in 12 L pots containing a sterile 3:1 sand/soil mix (15 replicates per treatment: two AMF genotypes and the control). Soil sterilisation was confirmed by sequencing soil samples before and after sterilisation, with large decreases in the diversity of bacterial and fungal DNA seen after sterilisation (Supplementary Fig. S1). Pots contained two types of pre-installed soil cores to simulate two different soil compartments: (i) the rhizosphere compartment (RC), with openings that allowed access to both AMF and plant roots; (ii) the bulk soil (SC), with openings covered with <32 μm mesh that allowed access by extraradical AMF hyphae, and other microbes, but excluded roots (Supplementary Fig. S2). After 70 days of maize growth the cores were removed, and it was confirmed there was no AMF colonisation in CTL treatments using microscopy of roots removed from RCs combined with mRNA sequence data from the root core (Supplementary Fig. S3). During this time, a stochastically assembled microbial community from the greenhouse environment was allowed to establish in all pots. The experiment was conducted in a greenhouse at 28°C with 60% relative humidity and a 12-h photoperiod. Pots were watered twice a week with 500 ml of tap water. For detailed information on plant, substrate, potting materials see Supplementary Notes S1.

### Taxonomic and transcriptome profiling of soil microbial communities in the rhizosphere and bulk soil compartments

The RC and SC were removed from 45 pots (15 replicates of each treatment) after 70 days of maize growth. To taxonomically profile the microbial communities, present in both soil compartments, we used Illumina paired-end 250 bp target amplicon sequencing of the ITS2 region for fungi using the primer pair fITS7-ITS4 [28, 29] and the V3-V4 region of the 16S rRNA gene for bacteria using the primer pair 347F-803R [30]. To investigate the transcriptional responses of AMF, plants and the metabolically active soil microbial community, we used a shotgun metatranscriptomic approach targeting soil total messenger RNA (mRNA) transcripts in both soil compartments. For full details about soil nucleic acids isolation, library preparation and sequencing see Supplementary Notes S2.

Microbial community taxonomic composition was assessed using quality filtered 16S rRNA and ITS sequences for bacteria and fungi, respectively. Sequences were quality filtered using bbtools v39.01 (https://sourceforge.net/projects/bbmap/), adapter trimmed using cutadapt v4.1 [31] and denoised into amplicon sequence variants (ASVs) using dada2 v1.24 [32] for the 16S rRNA amplicon or clustered at 97% similarity into operational taxonomic units (OTUs) for fungi. Taxonomic classifications were assigned to features using a Bayes classifier fitted with trained models on the target amplicon regions of the SILVA v138.1 [33–35] and UNITE v9 (dynamic all eukaryotes 25.07.2023) [36] databases for bacteria and fungi, respectively. All steps were implemented using *qiime2* pipeline v2022.8.0 [37] with feature count tables and taxonomic maps exported to phyloseq v1.38 [38] for downstream analysis. Amplicon raw sequence data were deposited in the NCBI Sequence Read Archive (SRA) under the project accession number PRJNA1288746.

Microbial mRNA transcript composition from each soil compartment was determined from the soil metatranscriptome. Sequences were first quality checked using FastQC (v0.11.9), keeping only reads with a quality score greater than 30 and sorted into ribosomal (rRNA) and non-ribosomal (non-rRNA) origin using SortMeRNA (v.4.0) [39]. Quality filtered non-rRNA read pairs were merged using PEAR (v0.9.8) [40] and aligned to the UniProtref90 database v2023.01 (https://www.uniprot.org) with Diamond v2.0.15 [41]. A 90% sequence similarity cut-off was set to filter for sequences most likely to be true matches. Two count tables were created using featureCounts v2.0.3 [42]. One containing all UniProt accessions across all samples and the second, to speed-up downstream computing, containing only UniProt accessions that were present in at least one third of the samples. All UniProt accessions matching to *Z. mays* or *R. irregularis* were removed prior to downstream analysis. Metatranscriptomic raw reads were deposited in deposited in the NCBI-SRA under the project accession number PRJNA1288746.

### Plant and arbuscular mycorrhizal fungi transcriptome read mapping

To filter transcriptome reads of maize and AMF origin from the soil metatranscriptome data, we used the STAR aligner v2.7.6a [43] to map all non-rRNA reads to *Z. mays* B73 reference genome v4 [43, 44] and to the *R. irregularis* DAOM 197198 reference genome (NCBI accession GCF_000439145.1), respectively. Maize reads were obtained only from the RC whereas AMF reads were obtained from both the RC and SC. In both cases, BUSCO [45] was used to check transcriptome completeness (Supplementary Fig. S4A and B) and featureCounts to create read count tables.

### Leaf nutrient measurements

At the time of core removal, leaf samples were taken for nutrient analysis. Half of the newest fully emerged leaf (defined as a leaf with a fully developed collar) on each plant was cut and dried in a drying chamber at 60°C for 5 days for later nutrient content analysis.

The leaf sample was ground to a fine powder using a ball mill and dissolved by means of a microwave-assisted digestion in nitric acid [46]. Briefly, the leaf sample was weighed to the nearest milligram and placed in a pressure vessel. Following the addition of 0.5 ml of deionized water and 5 ml of concentrated (65%) nitric acid, the sample was digested at 200°C for 30 min. After cooling, the solution was transferred to a 25 ml volumetric flask and diluted in deionized water prior to analysis for total element content on an inductively coupled plasma optical emission spectrometer (ICP-OES).

### Statistical analyses

All downstream analyses were conducted in R v4.1.0 [47]. Prior to ANOVA, normality and homogeneity of variances were checked using a Shapiro–Wilk test and Levene’s test respectively. A Tukey HSD test was used for post hoc comparisons. Prior to permutational multivariate analysis of variance tests (PERMANOVA) homogeneity of multivariate dispersions was tested with the betadisper function in the vegan package v2.6 [48, 49]. All Community dissimilarities were visualized using a non-metric multidimensional scaling (NMDS) ordination as implemented in phyloseq v1.38 package. For a detailed overview of all statistical analysis used see Supplementary Notes S3.

## Results

A total of 23 156 bacterial ASVs and 757 fungal OTUs were detected across all samples and the metatranscriptomic assembly yielded 2 363 990 genes, with large numbers of taxa and genes unique to each treatment. For an overview of the number of unique ASVs, OTUs and genes by treatment see Supplementary Tables S1-S3.

### Soil microbial communities differ between rhizosphere and bulk soil and in the presence of arbuscular mycorrhizal fungi

**Figure 1:**
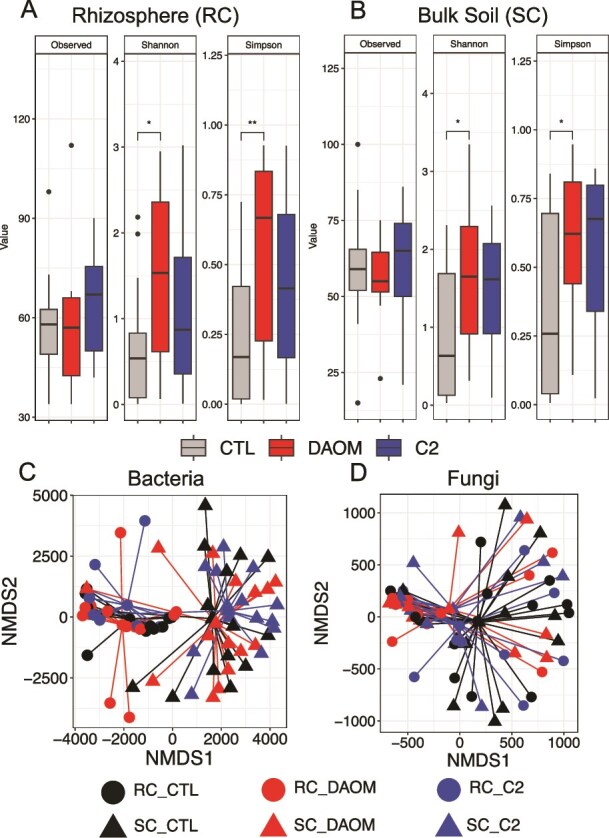
Alpha diversity and community composition of soil microbial taxa in rhizosphere and bulk soil under different inoculation treatments. (A and B) Alpha diversity indices of fungal communities in the rhizosphere (A) and bulk soil (B) under two AMF inoculation treatments and the non-inoculated control (CTL). Fungal diversity varied significantly among treatments, with stronger effects observed in the rhizosphere than in bulk soil. (C and D) Non-metric multidimensional scaling (NMDS) ordination plots showing community structure of bacterial taxa (C) and fungal taxa (D). Microbial communities clustered by soil compartment, indicating distinct rhizosphere and bulk soil assemblages. (A–D) Asterisks indicate significant differences between treatments (^*^P < .05, ^**^P < .01; Tukey HSD *post hoc* test).

There was an effect of the AMF DAOM on fungal community alpha diversity indices (Fig. 1A and B). Inoculation with DAOM resulted in significantly higher Shannon and Simpson indices in RC and SC compared to controls suggesting that the presence of this AMF genotype resulted in taxonomically richer and more even fungal communities (Fig. 1A and B). The same indices increased in the presence of C2 in both soil compartments although the differences were not statistically significant (Fig. 1A and B). Bacterial taxonomic richness and evenness did not increase across the two soil compartments in response to inoculation with either of the AMF genotypes (Supplementary Fig. S5). The composition of the bacterial community that established in the rhizosphere was significantly different from the bacterial community that established in the bulk soil (Fig. 1C and Table 1). The composition of the fungal community that established in the rhizosphere was significantly different from the community that established in the bulk soil after inoculation with both AMF genotypes (Fig. 1D and Table 2). The bacterial community composition within soil compartments was not altered following inoculation with either of the AMF genotypes (Fig. 1C and Table 1). Contrary to increases in taxonomic richness and evenness in the fungal community due to AMF inoculation, the community composition of fungal taxa did not shift in the presence of AMF in both compartments (Fig. 1D and Table 2).

**Table 1:** PERMANOVA results testing differences in bacterial community composition across inoculation treatments.

	Rhizosphere vs Bulk Soil					Rhizosphere					Bulk Soil				
	Df	SumOfSqs	*R^2^*	*F*	*P*adj	Df	SumOfSqs	*R^2^*	*F*	*P*adj	Df	SumOfSqs	*R^2^*	*F*	*P*adj
CTL	1	0.813	0.079	2.417	**.001**										
DAOM	1	0.916	0.089	2.737	**.001**										
C2	1	0.943	0.091	2.815	**.001**										
CTL-DAOM						1	0.366	0.037	1.073	.181	1	0.327	0.036	1.045	.222
CTL-C2						1	0.352	0.036	1.039	.282	1	0.296	0.033	0.943	.783
DAOM-C2						1	0.355	0.035	1.025	.324	1	0.304	0.034	0.990	.502

Significant values (*P*_adj_ < .05) are denoted in bold. (Df) degrees of freedom, (SumOfSqs) sums of squares, (*R*^2^) coefficient of partial determination, pseudo-F statistic (F), and (*P*_adj_) *P*-value adjusted for false discovery rate.

**Table 2:** PERMANOVA results testing differences in fungal community composition across inoculation treatments.

	Rhizosphere vs Bulk Soil					Rhizosphere					Bulk Soil				
	Df	SumOfSqs	*R^2^*	*F*	*P*adj	Df	SumOfSqs	*R^2^*	*F*	*P*adj	Df	SumOfSqs	*R^2^*	*F*	*P*adj
CTL	1	0.518	0.039	1.149	.089										
DAOM	1	0.537	0.041	1.195	**.015**										
C2	1	0.538	0.042	1.217	**.028**										
CTL-DAOM						1	0.480	0.039	1.122	.163	1	0.492	0.038	1.101	.234
CTL-C2						1	0.405	0.033	0.961	.520	1	0.404	0.031	0.906	.777
DAOM-C2						1	0.466	0.037	1.081	.214	1	0.424	0.033	0.965	.589

Significant values (*P*_adj_ < .05) are denoted in bold. (Df) degrees of freedom, (SumOfSqs) sums of squares, (*R*^2^) coefficient of partial determination, pseudo-F statistic (F), and (*P*_adj_) *P* value adjusted for false discovery rate.

### Differences in microbial gene transcription (excluding arbuscular mycorrhizal fungi) in rhizosphere and bulk soil in the presence and absence of *R. irregularis*

**Figure 2:**
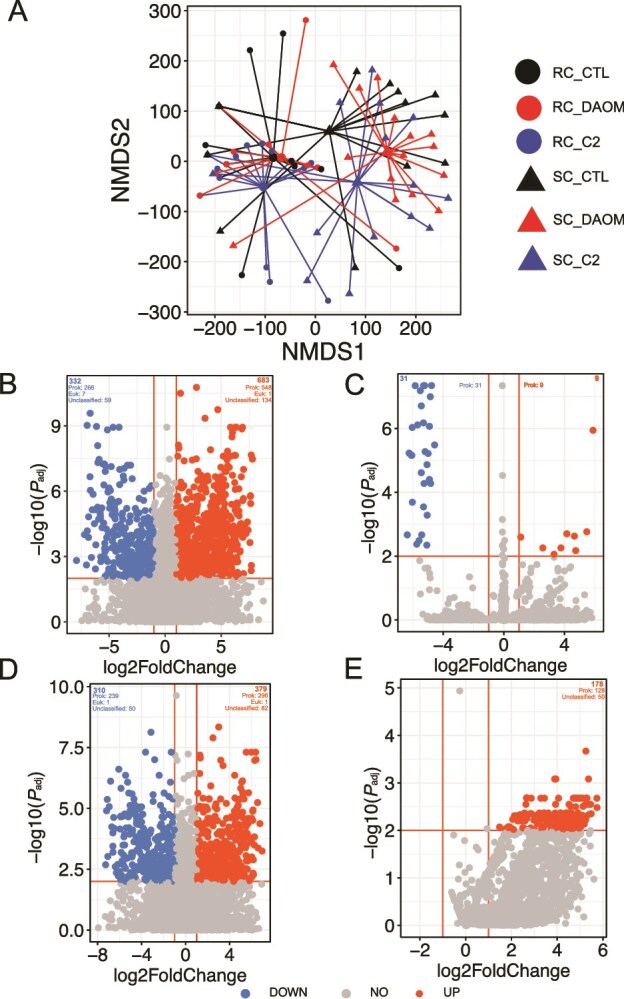
Differences in microbial gene expression between rhizosphere and bulk soil compartments. (A) Non-metric multidimensional scaling (NMDS) ordination of transcribed genes, showing clear separation between rhizosphere and bulk soil communities. (B and C) Volcano plots of differentially expressed genes in the rhizosphere (B) and bulk soil (C) for AMF inoculation with *Rhizophagus irregularis* DAOM compared to controls. (D&E) Volcano plots of differentially expressed genes in the rhizosphere (D) and bulk soil (E) for AMF inoculation with *Rhizophagus irregularis* C2 compared to controls. Genes significantly up- or down-regulated under inoculation are highlighted, with associated GO biological process terms provided in Supplementary Data 1.

Inoculation with the two AMF isolates did not significantly change transcribed mRNA richness or evenness within RC and SC compared to controls, suggesting that each AMF treatment and the control had a similar richness and evenness of mRNA transcripts (Supplementary Fig. S6). The composition of transcribed microbial mRNAs was significantly different between the rhizosphere and bulk soil (Fig. 2A and Table 3). The AMF genotype DAOM significantly influenced the composition of transcribed genes from the microbiome in the bulk soil (Fig. 2A and Table 3). No significant effect on the composition of transcribed genes was seen in the rhizosphere following AMF inoculation, emphasising the importance of AMF on the microbiome in soil inaccessible to roots. These data would suggest AMF influence the transcription of given genes in the bulk soil but not on overall gene richness or evenness in the bulk soil.

**Table 3:** PERMANOVA results testing differences in the composition of microbiome-derived mRNA transcripts across treatments.

	Rhizosphere vs Bulk Soil					Rhizosphere					Bulk Soil				
	Df	SumOfSqs	*R^2^*	*F*	*P*adj	Df	SumOfSqs	*R^2^*	*F*	*P*adj	Df	SumOfSqs	*R^2^*	*F*	*P*adj
CTL	1	0.830	0.141	4.592	**.012**										
DAOM	1	1.674	0.321	13.250	**.001**										
C2	1	1.129	0.223	8.053	**.001**										
CTL-DAOM						1	0.156	0.036	1.045	.318	1	0.377	0.089	2.724	**.040**
CTL-C2						1	0.209	0.049	1.438	.134	1	0.225	0.048	1.400	.199
DAOM-C2						1	0.139	0.032	0.919	.465	1	0.092	0.031	0.901	.467

Significant values (*P*_adj_ < .05) are denoted in bold. (Df) degrees of freedom, (SumOfSqs) sums of squares, (*R*^2^) coefficient of partial determination, pseudo-F statistic (F), and (*P*_adj_) P value adjusted for false discovery rate.

A differential expression analysis of GO terms on annotated genes revealed 683 upregulated and 332 downregulated genes in the rhizosphere of the DAOM treatment and nine upregulated and 31 downregulated in the bulk soil, compared to the control (Fig. 2B and C). In the C2 treatment, 379 genes were upregulated and 310 downregulated in the rhizosphere, and 178 upregulated and no downregulated in the bulk soil compared to the control (Fig. 2D and E). Almost all these genes were of prokaryotic origin, based on UniProt assigned taxonomy of gene predictions. Genes related to B-vitamin biosynthesis were consistently upregulated in the rhizosphere when AMF were present despite AMF possessing no known genes encoding for b-vitamin biosynthetic pathways [50, 51] (Fig. 2B and D and Supplementary data 1). Full lists of differentially transcribed microbial genes in the RC and SC of each of the treatments versus the controls are available as Supplementary data 1.

### Arbuscular mycorrhizal fungi gene transcription differed between the rhizosphere and bulk soil

**Figure 3:**
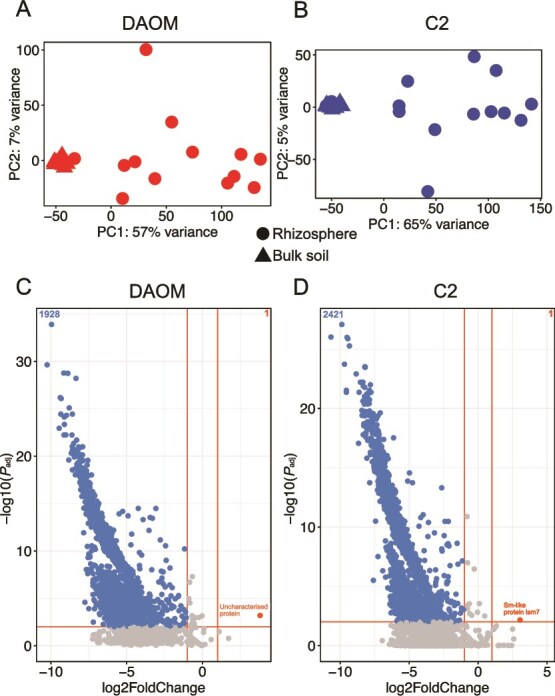
Profiles of AMF gene expression in rhizosphere and bulk soil. (A and B) Principal component analysis (PCA) of normalized, log2-transformed gene expression for *Rhizophagus irregularis* DAOM (A) and *Rhizophagus irregularis* C2 (B). Axes indicate the percentage of variation explained by each principal component. PERMANOVA tests confirmed significant differences in AMF gene composition between rhizosphere and bulk soil for both genotypes (*P* < .001). (C and D) Volcano plots of differentially expressed AMF genes in bulk soil versus rhizosphere for DAOM (C) and C2 (D). Protein names of the few genes upregulated in bulk soil are highlighted in red.

There were significant differences in transcription profiles of both AMF genotypes between the bulk soil and rhizosphere (Fig. 3A and B). Furthermore, expression profiles of the two genotypes were more similar in the bulk soil, whereas in the rhizosphere there was more variability in gene transcription profiles within genotypes (Fig. 3A and B). As expected, given the gene transcription required to communicate with the plant host, we found large differences in expression levels of AMF genes between the rhizosphere and bulk soil with only one gene per genotype being upregulated in the bulk soil versus the rhizosphere (Fig. 3C and D).

### Bacterial taxa are linked to the presence of arbuscular mycorrhizal fungi and playing a role in plant phosphorus uptake

**Figure 4:**
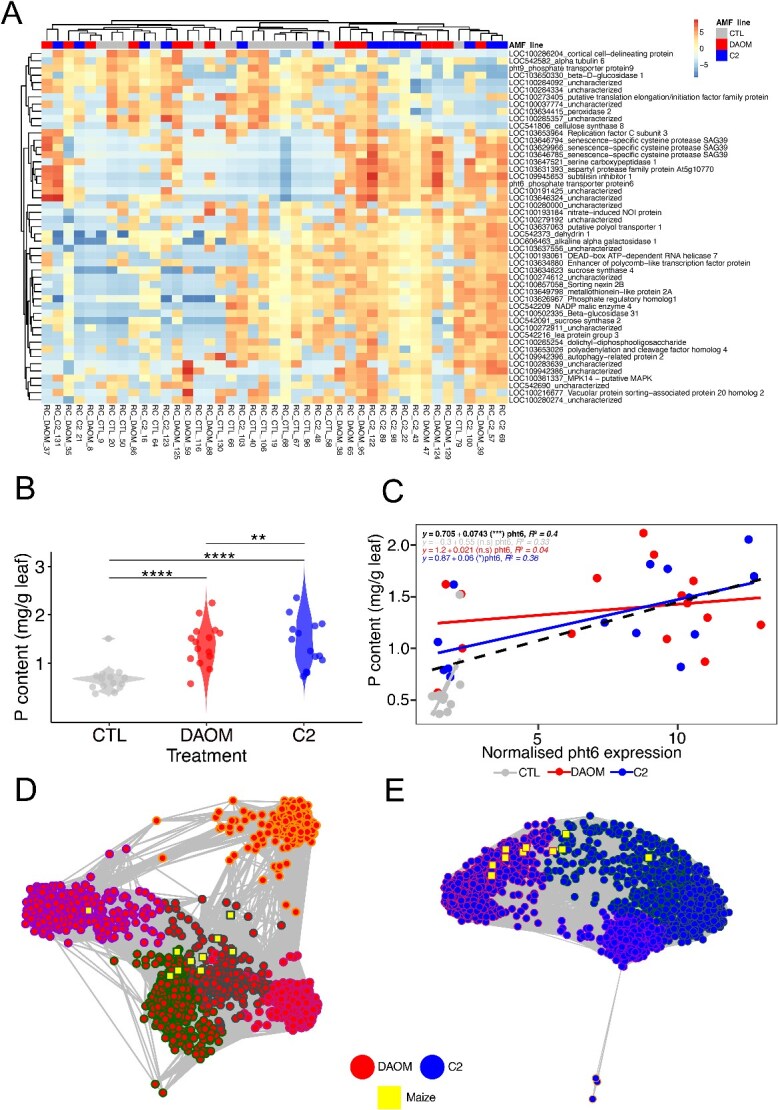
Maize transcriptional responses to AMF inoculation and their relationship with leaf phosphorus content. (A) Heatmap of the 50 most highly transcribed maize genes differing among treatments. Expression values are shown as Z-scores from DESeq2-normalised, log2-transformed counts. (B) Leaf phosphorus (P) content under control (CTL), Rhizophagus irregularis DAOM, and C2 inoculation treatments. One-way ANOVA followed by Tukey’s post hoc test revealed significant differences among all pairwise comparisons (asterisks indicate significance on the plot). (C) Linear regression between maize leaf P content and *pht6* expression in roots across all samples, showing a positive association. (D and E) Co-occurrence network analyses of AMF transcribed genes with 10 selected maize genes in the rhizosphere for DAOM (D) and C2 (E). Node-level ANOVA tests indicated significant differences in cluster membership across most network metrics (degree, average path length, clustering coefficient), with betweenness centrality significant only in DAOM. Red and blue nodes represent DAOM and C2 genes, respectively; yellow square nodes represent maize genes; outer circle colours denote gene clusters.

Hierarchical clustering of the distance matrix of the 50 most variable maize genes (based on Euclidean distances) confirmed maize gene expression clustered by AMF genotype and indicated that certain maize genes were specific to the symbiosis with AMF compared to controls (Fig. 4A). To further assess these relationships in reduced dimensional space, we performed a PCA restricted to the same 50 genes. The PCA confirmed that the primary axes of variation separated samples according to AMF colonisation, consistent with the patterns observed in the heatmap (Supplementary Fig. S7). Based on the heatmap clustering and PCA, maize genes associated with AMF colonisation were further assessed across the two genotypes of *R. irregularis* (DAOM and C2). These genes, including *pht6* (a phosphate transporter in maize roots activated in the mycorrhizal symbiosis [52]), were also found to be differentially expressed in AMF treatments compared to controls (Supplementary Fig. S8). We found higher leaf P content in AMF treatments (Fig. 4B) and a positive linear correlation between plant leaf P and *pht6* expression across AMF inoculated samples which was significant for the genotype C2 (Fig. 4C). Taken together these results suggests active delivery of P through *pht6* in roots colonised by AMF. Furthermore, co-occurrence network analysis based on these 10 maize transcribed genes and either DAOM or C2 transcribed genes in the rhizosphere showed high network modularity. For the DAOM genotype, ANOVA tests revealed highly significant between-cluster differences across all node-level metrics (degree: *F* = 1107, *P* < .001; average path length: *F* = 6734, *P* < .0001; clustering coefficient: *F* = 482.4, *P* < .001; betweenness centrality: *F* = 8.2, *P* < .001.For the C2 genotype, cluster membership significantly affected degree (*F* = 719.2, *P* < .001), average path length (*F* = 1291, *P* < .001), and clustering coefficient (*F* = 436.2, *P* < .001), but not betweenness centrality (*F* = 2, *P* = .089). Furthermore, for the C2 genotype three genes appeared distinct from the primary cooccurrence module. Since these genes encoded for an uncharacterised protein, polyubiquitin and Ras protein Rab6 and given their peripheral placement they likely play a general cellular or regulatory role i.e. not strongly synchronised with the majority of C2 transcriptional activity in the rhizosphere as thus appear as distinct in the network analysis. For maize, the gene *SAG39*, a senescence-specific cysteine protease was in distinct clusters from other maize symbiosis-related genes for both the DAOM and C2 genotypes. These results would suggest that AMF genes could be involved in regulating expression of maize genes involved in symbiosis and vice versa although significant further work would be required to confirm this.

**Figure 5:**
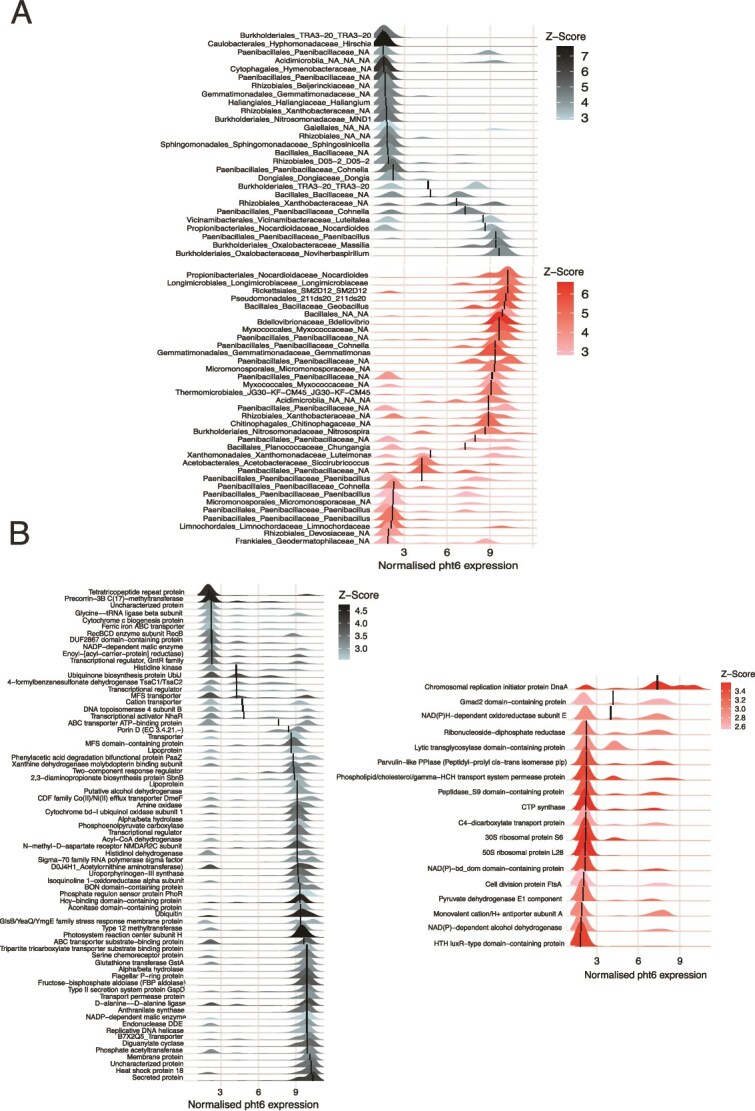
Microbial associations with maize *pht6* expression as identified by TITAN2 analysis. (A) Bacterial taxa showing significant positive (red) or negative (blue) responses to *pht6* transcript abundance in maize roots (for fungal associations see Supplementary Fig. S8). (B) Microbial mRNA transcripts showing positive (red) or negative (blue) responses to *pht6* transcript abundance in maize roots. Positive associations indicate taxa/transcripts enriched when *pht6* expression is high, whereas negative associations indicate enrichment when *pht6* expression is low. Colour intensity reflects the strength of the association (TITAN2 z-scores), and only taxa/transcripts meeting significance thresholds are shown.

Based on these data, we used *pht6* as a proxy for describing a functional AM symbiosis in maize and to identify the microbial taxa associated with the symbiotic function in maize roots. The abundance of 27 bacterial taxa decreased in response to increases in *pht6* expression whereas 35 bacterial taxa increased in abundance (Fig. 5A). An increase in the abundance of three fungal taxa was associated with activation of the *pht6* gene and there were no fungal taxa that decreased in abundance (Supplementary Fig. S8). Using the microbiome transcriptional data, we found 84 mRNA UniProt annotated transcripts that were negatively associated with *pht6* and 19 mRNA UniProt annotated transcripts that were positively associated with *pht6* expression (Fig. 5B).

## Discussion

Our findings show that AMF alter bacterial gene expression without shifting community composition, significantly increase fungal richness and evenness, stimulate microbial B-vitamin biosynthesis, and increase the abundance of rhizosphere bacterial taxa linked to elevated plant leaf phosphorus levels. Differences in the composition of transcribed bacterial mRNAs in the bulk soil with AMF compared to when no AMF were present but no changes in bacterial taxonomic composition can be explained in several ways. 16S rRNA taxonomic profiling of the bacterial community is a relatively low-resolution technique that cannot distinguish between closely related strains within the same taxon. Functional traits, including metabolic activity, stress responses, and nutrient acquisition strategies, often vary substantially at the strain level [53]. It is therefore plausible that AMF selectively influenced the activity of strains within broadly defined taxa, leading to marked changes in gene expression without detectable shifts in overall taxonomic composition. There are also several possible biological explanations. Firstly, AMF may have direct interactions with the soil microbial community to stimulate specific functions. Functional redundancy may mean that many microbial taxa have overlapping functions and, therefore, AMF may be influencing the existing community rather than recruiting new members. Recent work looking at AMF small RNAs suggest that they can alter plant gene transcription and signalling cascades [54] and it is likely that similar small RNAs can alter bacterial gene transcription as well [55]. There is also evidence to suggest that AMF metabolites may act as signalling molecules to surrounding microbes [20]. Secondly, AMF may indirectly affect gene transcription in the soil microbial community by altering the rhizosphere and bulk soil environment through exudates, modifications to root physiology, and nutrient gradients which in turn would shift the metabolic activity of microbes [56].

We found that AMF increased the fungal community evenness and richness in both the rhizosphere and bulk soil based on ITS amplicon profiles. Prior research has focused on AMF-bacteria interactions [57] with less attention paid to AMF fungal interactions [10]. Our results emphasise the importance of AMF interactions with other soil fungi. The greater fungal richness and evenness we observed in the bulk soil may be explained by a priming effect of AMF supplied carbon which can stimulate fungal saprotrophic communities in the presence of organic matter [58]. Additionally, AMF colonisation of the rhizosphere is known to alter the plant root metabolome which has been shown to affect other root fungi [59] and, indeed, our data showed significant alterations to the maize root transcriptome after AMF colonisation.

There is evidence supporting that the effect of AMF on the richness and evenness of rhizosphere fungal and bacterial taxa is dependent on the host plant [60] as well as the AMF genotype [12, 61]. Our results also indicated AMF genotype specific effects, with only DAOM causing a significant increase in fungal species richness and evenness in the rhizosphere and bulk soil. However, both genotypes caused a significant change to fungal community composition in the rhizosphere compared to bulk soil. It is likely the two genotypes fill different ecological niches. In natural soils, many AMF genotypes coexist on one plant filling a specific ecological niche [62]. Assuming AMF hyphae are exuding carbon into the rhizosphere and bulk soil for microbial uptake, as has been established [7], differences in fungal growth strategies between the two genotypes would explain the differences in interactions with the microbial community.

Interestingly, amongst the genes that were upregulated by both AMF genotypes in the rhizosphere and by C2 in the bulk soil were genes involved in B vitamin biosynthetic pathways. *R. irregularis* lacks several genes in B vitamin biosynthetic pathways in contrast to the presence of these genes in other fungi [50, 51]. Previous research has found a mutually beneficial relationship in vitro whereby vitamin B1 can be transferred to the filamentous fungi *Aspergillus nidulans* from a strain of *Bacillus subtilis* growing in association with the fungal hyphae and that vitamin B1 biosynthesis related genes were induced in the bacteria [63]. Addition of exogenous vitamin B2 to the rhizosphere significantly increased AMF colonisation [64] and it is possible that AMF also stimulate soil microbes to produce these molecules. For instance, AMF are known to exude a wide variety of carbon compounds into the soil through their hyphae [16, 65] and it has been shown that similar carbon compounds can induce production of vitamins by rhizosphere microbes in vitro [66]. Furthermore, AMF are known to accumulate vitamin B1 in spores, suggesting it is needed for hyphal growth and not produced endogenously [67].

Using plant leaf P data, we found that inoculation with either AMF genotype increases plant P uptake compared to the controls. This can be explained in part by AMF induced changes in maize gene expression whereby we find evidence for a direct pathway and P uptake via AMF colonisation. This is in line with previous research which has shown plants preferentially use specific phosphate transporters in symbiosis with AMF [68–70]. In general, we found 10 genes significantly associated with AMF colonisation. Co-occurrence network analysis revealed a clear modular organisation of maize and AMF genes in the rhizosphere. Maize symbiosis-related genes clustered together with specific AMF transcripts, suggesting a coordinated cross-kingdom transcriptional module that likely underlies the establishment and maintenance of the symbiotic interaction. However, the maize gene *SAG39*, which encodes a senescence-specific cysteine protease involved in programmed cell death, formed a separate cluster in both DAOM and C2 networks. This separation indicates that *SAG39* is regulated independently from the main symbiotic program and may represent a transcriptional module associated with arbuscule turnover or symbiosis termination. Together, these results highlight the existence of distinct regulatory modules within the maize-AMF interaction: a core maintenance module linking host and fungal gene expression, and a separate cell-death-related module that may act to limit or terminate symbiotic activity. Unfortunately, the majority of AMF transcripts in the network remain uncharacterised but our results provide clues about which AMF genes are invovled in maintenance (e.g. nutrient exchange) of the symbiosis and which genes are involved in turnover of the symbiosis.

Our data tentatively link maize symbiosis-related genes to changes in taxonomy and gene expression in the rhizosphere microbiome, with the caveat that the plant and AMF effect cannot be separated as there is likely reciprocal gene regulation. Plant genes are linked to the recruitment of specific microbial communities in the rhizosphere [71, 72] yet AMF cause major transcriptional changes in plant genes [52] which in turn shape the rhizosphere microbiome. Using the maize gene *pht6* which is activated under AMF colonisation [52] we showed that the abundance of certain bacterial taxa increased in response to activation of *pht6* and certain bacterial taxa decreased in abundance. Among the positive responders were 11 taxa in the family *Paenibacillaceae*, four of which could be identified at genus level as *Paenibacillus*. These are well known plant growth promoting bacteria [73], and have previously been found in association with AMF [74, 75]. Combined inoculation of *Paenibacillus* with AMF also significantly increases shoot P content [76]. The taxa are known to possess chitinolytic capabilities and mineralise dead fungal hyphae in the rhizosphere, increasing nutrient turnover and availability [17, 77]. In our study we also found increases in abundances of the chitinolytic *Chitinophagaceae* and bacterivorous *Bdellovibrionaceae* in response to *pht6* activation. Both taxa have been shown to contribute to mineralisation of fungal hyphae in the soil alongside *Paenibacillus* [77, 78] which would release the nutrients contained in fungal hyphae (e.g. N and P) into the soil. Among the 19 microbial genes that were associated with *pht6* expression we did not find any with an obvious role in chitin degradation. Instead, most of these transcripts relate to bacterial growth and metabolism and likely capture the proliferation of the taxa associated with AMF rather than their specific function. Interestingly, amongst the microbial transcripts negatively responding to *pht6* expression is *Phosphate regulon sensor protein (PhoR)*. This gene is activated in P-limiting environments in bacteria [79] and can be regulated by different C sources [80]. This, in combination with the increases in abundance of chitin degrading taxa, would suggest increased rhizosphere P comes from degradation of P-containing AMF hyphae, although stable isotope probing would be required to confirm this.

## Conclusion and future perspectives

Overall, our study has provided insights into the microbial dynamics in the rhizosphere and bulk soil in the presence of AMF in a single pot system. Specifically, our results associate increases in plant P through AMF colonisation with changes in maize gene expression and changes in the abundance of certain bacterial taxa in the rhizosphere. Additionally, we explore soil microbial mRNAs differentially transcribed in the rhizosphere and bulk soil in the presence of AMF which provide insights into the functional interplay between AMF and the soil microbiome. For instance, we demonstrate microbial functions relating to vitamin biosynthesis are upregulated in the presence of AMF. In future, it will be crucial to establish connections between AMF gene expression and alterations in soil microbial community function and assembly to complement the indirect effects we observed after AMF-induced changes to host plant gene expression. We believe our results emphasise the role of feedback loops between plant and soil microorganisms and their importance in nutrient uptake along the plant-fungal-soil microbial axis.

## Supplementary Material

Wright_et-al_SuppInfo_final_wraf282

Wright_et-al_SuppData_final_wraf282

## Data Availability

Raw sequence data are available under NCBI BioProject PRJNA1288746. Metatranscriptomic assessions are from SRR34437684 to SRR34437881, amplicon 16S rRNA SRR34437672 to SRR34437682 and SRR34437729 to SRR34437878 and amplicon ITS are from SRR34437693 to SRR34437727.
