# Supplementary material for: Mycorrhizal control of microbial gene transcription and taxonomic composition in the rhizosphere and bulk soil
Source: ISME J. 2025 Dec 19;19(1):wraf282. doi: 10.1093/ismejo/wraf282 (PMC12766751; doi:10.1093/ismejo/wraf282)
Supplement: Wright_et-al_SuppInfo_final_wraf282 [file wright_et-al_suppinfo_final_wraf282.docx]

**Supplementary Information**

**Title**

Mycorrhizal control of microbial gene transcription and taxonomic composition in the rhizosphere and bulk soil

**Authors**

Fergus Wright^1^, Stéphanie Grand^2^, Ian Sanders^1^ & Ricardo Arraiano-Castilho^1^

**Authors affiliations**

1 - Department of Ecology and Evolution, University of Lausanne,

Lausanne 1015, Switzerland

2 - Institute of Earth Surface Dynamics, University of Lausanne, 1015, Switzerland

**Correspondence**

Ricardo Arraiano Castilho - [ricardo.m.castilho@gmail.com](mailto:ricardo.m.castilho@gmail.com)

Department of Ecology and Evolution, University of Lausanne,

Lausanne 1015, Switzerland

**ORCID Ids**

Fergus Wright – <https://orcid.org/0009-0009-2050-0436>

Stéphanie Grand - <https://orcid.org/0000-0002-0239-3536>

Ian Sanders – <https://orcid.org/0000-0002-9591-8214>

Ricardo Arraiano Castilho - <https://orcid.org/0000-0001-8465-5909>

**Supplementary notes S1**

**Plant and substrate material**

Seeds of *Zea mays* (B73) were surface sterilised and left on moistened filter paper in petri dishes to germinate in the dark at 25 degrees for 4 days. Seeds had been kindly supplied from the University of Basel and had received no prior seed treatment or coating. Surface sterilisation was as follows. Seeds were pre-treated with 70% ethanol for 3 minutes with gentle stirring, decanted, and then sterilized with 7% sodium hypochlorite (approximately 1 mL per seed) for 15 min. Seeds were washed five times with sterile water (2 minutes per wash) and soaked in sterile water for 12 h. Sterilized seeds were placed on autoclaved filter paper in glass Petri dishes with 2 mL sterile water and allowed to germinate. Seeds typically germinated within 48 h and were ready for planting within 3–4 days.

Clay loam topsoil from a construction site located at the University of Lausanne was twice sterilised using a Sterilo machine (Harter ERDDÄMPFER, <https://sterilo.de>). The internal temperature of the soil (centre of the Sterilo machine) was raised to 110 degrees for 90 minutes and mixed between the runs to ensure homogenous sterilisation. After sterilisation the sterile soil was mixed (3:1 ratio) with 1.7mm quartz sand that had previously been sterilised once at 110 degrees for 90 minutes using the Sterilo machine. The resultant substrate had the following chemical properties; pH 8.0, Inorganic C [%] 0.5, Organic C [%] 1.1, Total N [%] 1.1, C/N ratio 4.7, Exch Al [cmol/kg] 0.01, Exch Ca [cmol/kg] 18.66, Exch K [cmol/kg] 0.7, Exch Mg [cmol/kg] 0.4, Exch Na [cmol/kg] 0.2, Exch P [cmol/kg] 0.06, CEC [cmolc/kg] 39.1, Base Saturation [%] 99.9, Al tot [mg/kg soil] 7453, Ca tot [mg/kg soil] 11973, Cu tot [mg/kg soil] 10, Fe tot [mg/kg soil] 6558, K tot [mg/kg soil] 1947, Mg tot [mg/kg soil] 2238, Mn tot [mg/kg soil] 222, Na tot [mg/kg soil] 255, P tot [mg/kg soil] 296, Si tot [mg/kg soil] 227, Zn tot [mg/kg soil] 25.

**Creation of compartmentalised pot mesocosms**

The rhizosphere compartment (RC) allowed access to plant roots, AMF hyphae and the soil microbiome. It was created by cutting a hole along one side of a falcon tube. The bulk soil compartment (SC) excluded plant root access but allowed access by AMF hyphae and the soil microbiome and was the same as the RC compartment except a mesh (<32µm) was glued over the hole. Twelve litre pots were filled with substrate and the 2 modified falcon tubes, filled with the same substrate, were inserted in each pot. Pregerminated *Z. mays* seeds were planted at the centre of each pot and the pots were arranged in a randomised block design in the greenhouse at 28 degrees with 60% relative humidity and a 12-hour photoperiod. Pots were watered twice a week with 500ml of tap water.

**Inoculation of AMF**

Spores of *Rhizophagus irregularis* DAOM 197198 were provided in suspension by a commercial producer (Symbiom, Sázava 170, 56301 Lanškroun, Czech Republic) having been cultured *in vitro* in root organ cultures. Spores of *R. irregularis* C2 were produced from our own lab collection using root organ cultures and prepared as spore suspensions for inoculation of plants. Approximately 500 spores of each AMF isolate were used to inoculate *Z. mays* plants in AMF treatments. The spore suspension was pipetted evenly around the base of *Z. mays* plants one week after they had been planted. Controls were inoculated in the same way but using only sterile Mili-Q water.

**Supplementary notes S2**

**Soil DNA and RNA isolation, library preparation and sequencing**

Fifteen replicates of the RC and SC were used for both DNA and RNA extractions. Soil total DNA and RNA was isolated using the DNeasy PowerSoil Pro Kit (Qiagen, Hilden, Germany) and Soil total RNA purification Kit (Norgen Biotek, Thorold, Canada) respectively following their standard protocols.

For amplicon sequencing both library preparation and sequencing were conducted at Génome Québec (Montréal, Canada) using Illumina MiSeq v3, PE-250 bp platform following their metabarcoding (16S, 18S, ITS) procedure. For fungi we targeted the ITS2 region using the primer pair fITS7-ITS4 [8, 9] and for bacteria we targeted the V3-V4 region of the 16S rRNA gene using the primer pair 347F-803R [10]. All PCRs were performed in three replicates per sample (25ul each) and pooled before library preparation using 0.02 U/ul FastStart High Fi 5U-ul (Roche), 1X PCR Buffer with 18 mM MgCl_2_ (Roche), 5% Dimethyl Sulfoxide (Roche), 0.2 mM Deoxynucleotide (dNTP) Solution Mix 10mM (New England Biolabs), 0.6 uM of each primer pair and final volume adjusted with ultra pure H_2_O. Thermal cycling conditions for the ITS2 region (fITS7-ITS4) included an initial denaturation 96 °C for 15 min; 33 cycles of denaturation for 30s at 96°C, annealing for 30s at 52°C, elongation for 1min at 72°C; final elongation at 72 °C for 10 min. For the V3-V4 region of the 16S rRNA gene (347F-803R), an initial denaturation 94 °C for 2 min; 26 cycles of denaturation for 30s at 94°C, annealing for 30s at 55°C, elongation for 1min at 72°C; final elongation at 72 °C for 10 min. Indexing of each PCR pool was conducted using 0.025 U/ul FastStart High Fi 5U-ul (Roche), 1X PCR Buffer without MgCl_2_ (Roche), 1.8mM of MgCl_2_ (Roche), 5% Dimethyl Sulfoxide (Roche), 0.2 mM Deoxynucleotide (dNTP) Solution Mix 10mM (New England Biolabs), 2 ul of each index at 2 uM and final volume adjusted to 20ul with ultra pure H_2_O. Thermal cycling conditions for indexing were: initial denaturation 95 °C for 10 min; 15 cycles of denaturation for 15s at 95°C, annealing for 30s at 60°C, elongation for 1min at 72°C; final elongation at 72 °C for 3 min for both regions. All PCR products were puryfied with sparQ PureMag Beads (Quantabio) following manufacture’s protocol.

Metatranscriptomic library preparation and sequencing were conducted at the Genomic Technologies Facility (GTF) at the university of Lausanne. Sequencing libraries were prepared differently depending on the core types. For cores containing roots (RC), both bacterial ribosomal RNA (rRNA) and plant rRNA depletion kits were used (Qiagen QIAseq FastSelect – 5S/16S/23S Kit and Qiagen QIAseq FastSelect –rRNA Plant Kit). For cores without roots (SC) only bacterial rRNA depletion was conducted using the Qiagen QIAseq FastSelect – 5S/16S/23S Kit. Paired-end sequencing (150bp) was conducted using an Illumina NovaSeq 6000 sequencing platform with TruSeq Stranded Library Preparation.

Supplementary Note S3

**Soil microbial diversity and composition**

To test whether soil microbial taxonomic richness and composition were influenced by the presence and identity of AMF, we calculated alpha diversity metrics for bacterial and fungal communities, including observed richness, Shannon, and Simpson indices, across inoculation treatments and soil compartments. Raw count data was normalised using Hellinger transformation. Differences in community composition were assessed by first computing a Bray-Curtis dissimilarity matrix and with a PERMANOVA test as implemented in pairwise.adonis() function from pairwise.adonis R package v0.4 (a wrapper for multilevel pairwise comparison using adonis2 from package vegan that returns adjusted p-values [1,7]). Adjusted *P*-values were calculated using the “fdr” method to control for the expected proportion of false discoveries amongst the rejected hypotheses. Two sets of PERMANOVA tests were performed: (i) differences between rhizosphere and bulk soil for each inoculation treatment (DAOM, C2, CTL) and (ii) differences among inoculation treatments within each soil compartment.

**Microbial functional composition**

To assess differences in the functional composition of the soil microbiome, we analysed mRNA transcripts annotated with UniProtref90 and associated Gene Ontology (GO) biological process terms. Raw count data was normalised using Hellinger transformation. Pairwise Bray-Curtis dissimilarities were calculated and used in PERMANOVA tests like the taxonomic analyses: (i) between soil compartments for each treatment, and (ii) among inoculation treatments within each compartment. Differential expression analysis was conducted with DESeq2 v1.34.0 [2] to identify AMF-driven changes in microbial gene expression within each compartment.

**Plant and AMF transcriptional responses**

Differential expression analysis (DESeq2, v1.34.0) was also applied to AMF and maize mRNA reads to examine transcriptional responses across AMF genotypes, controls, and soil compartments. Principal component analysis (PCA) of normalized log2-transformed data was used to visualize variation in gene expression. Pairwise Euclidean distances between treatments were calculated from these data to assess differences in expressed genes. For AMF, distance matrices were used to test DAOM vs. CTL and C2 vs. CTL within each compartment, and to generate heatmaps of the 50 genes with the highest variance. For maize, to visualise the expression patterns of the most informative transcripts, we selected the top 50 genes with the highest variance across samples. The variance-stabilized expression values of these genes were used to generate both a heatmap and an ordination to assess clustering patterns among samples. For PCA, the matrix of expression values was transposed to obtain samples as rows and genes as columns, and principal component analysis was performed with the prcomp() function in R. The phosphate transport gene PHT6 was found to be associated with AMF colonization and used for subsequent analyses.

**Co-occurrence network analysis**

To investigate interactions between maize genes and AMF gene expression, we performed separate co-occurrence network analyses for each AMF genotype using igraph (v2.1.2) in R. Pearson correlation coefficients were calculated between genes, and a threshold of 0.75 defined edges in the undirected, weighted network. Community detection was performed with the Louvain algorithm, and node-level metrics (degree, average path length, clustering coefficient, betweenness centrality) were calculated. Differences in these metrics between clusters were evaluated using one-way ANOVA.

**Linking microbial community function to plant phosphorous uptake**

Leaf phosphorus (P) content was compared among inoculation treatments using one-way ANOVA followed by Tukey’s HSD post hoc test. Differentially upregulated maize genes in both AMF treatments were identified, with phosphate transporter 6 (*pht6*) selected as a proxy for AMF-mediated P delivery [3, 4]. Threshold Indicator Taxa Analysis (TITAN2; [5]) was applied to relate *pht6* expression to bacterial and fungal community composition (amplicon sequencing counts) and to microbial functional gene expression (UniProt-annotated metatranscriptomic reads), following standard protocols [6]. Prior to running TITAN2, raw PHT6 read counts were normalised using the DESeq2 variance-stabilising approach, and for the amplicon sequencing data counts were normalised using the Hellinger transformation.

Supplementary Table S1. Unique bacterial ASVs by treatment

Supplementary Table S2. Unique fungal OTUs by treatment

Supplementary Table S3. Unique UniProt annotated genes by treatment

**Supplementary Figure S1. Alpha diversity measures of soil samples taken before and after sterilisation**

(A) Bacterial alpha diversity measures before (SB), after one round (SB1), and after a second round of sterilisation (SB2). (B) Fungal alpha diversity measures before (SB), after one round (SB1), and after a second round of sterilisation (SB2).

**Supplementary Figure S2. Experimental design and pot system**

Experimental design illustrating treatments and two types of soil cores used in the experiment (rhizosphere core (RC) and bulk soil core (SC).


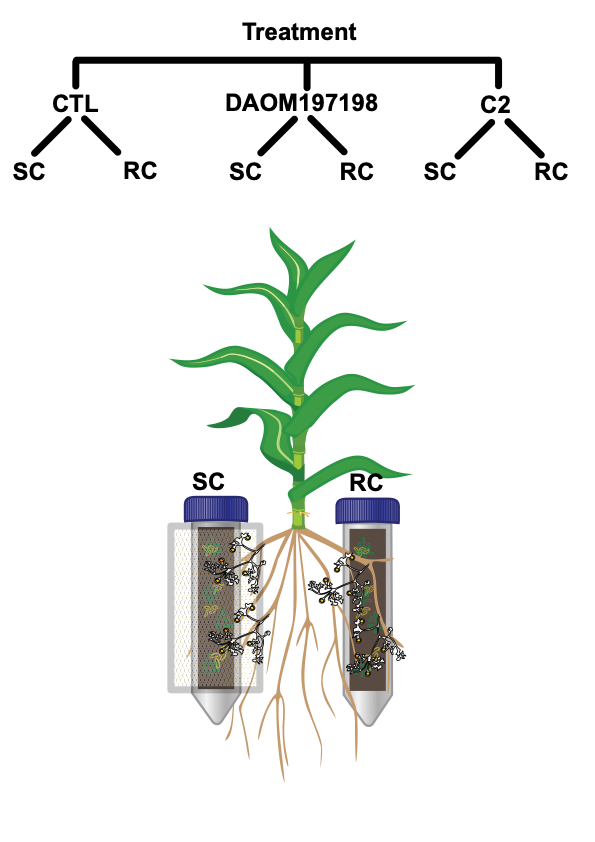


**Supplementary Figure S3. AMF colonisation check**

****(A) Root microscopy of AMF colonisation from roots taken from the RC at the time of removal. (B) The percentage of total metatranscriptomic reads assigned to the reference *R. irregularis* DAOM 197198 genome across the rhizosphere (RC) and bulk soil (SC).

**Supplementary Figure S4. BUSCO analysis of maize and AMF transcriptome**

Benchmarking Universal Single-copy orthologs (BUSCO) bar plots indicating complete and single-copy, complete and duplicated, fragmented and missing orthologs for the maize transcriptome (A) and the AMF transcriptome (B). In (B) the AMF transcriptome was assessed across both the rhizosphere (RC) and bulk soil (SC).


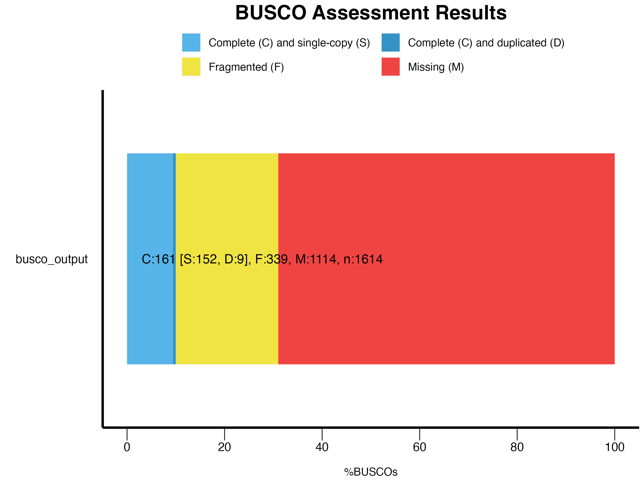

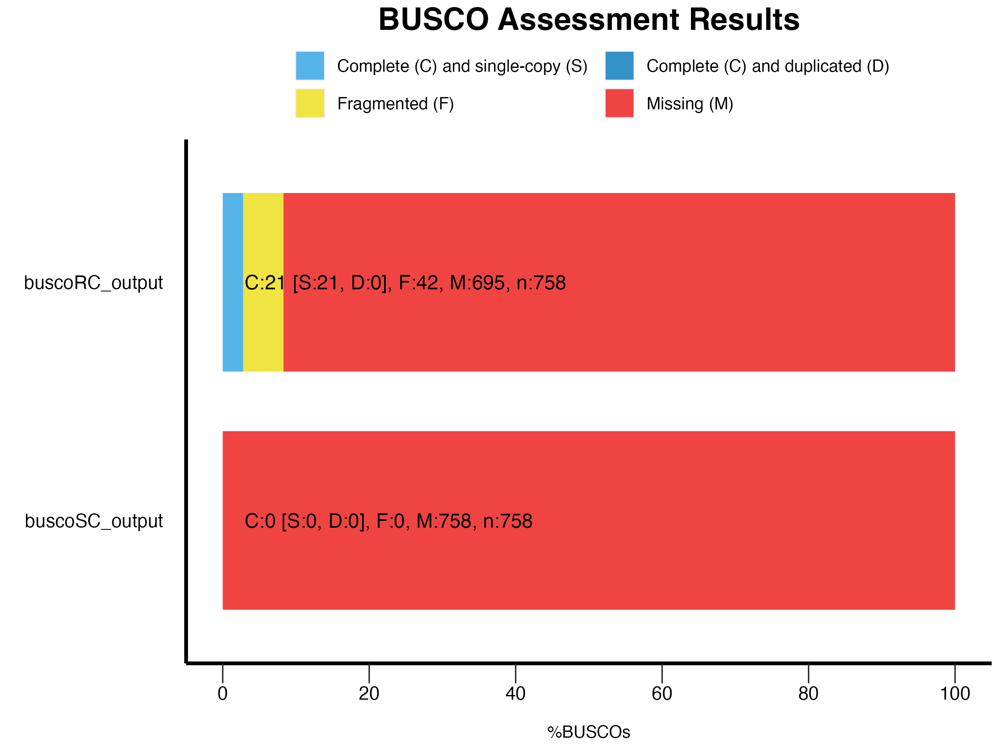


A

B

Maize

AMF

Supplementary Figure S5. Soil bacterial alpha diversity across cores

Alpha diversity indices of soil bacterial communities with the two AMF inoculation treatments (isolates DAOM and C2) and the non-inoculated control (CTL) in the rhizosphere (A) and bulk soil (B).

Supplementary Figure S6. Alpha diversity of mRNA transcripts across cores

Alpha diversity indices of soil bacterial and fungal mRNA transcripts with the two AMF genotypes in the rhizosphere (A) and bulk soil (B).

Supplementary Figure S7. PCA of samples based on expression of maize genes with highest variance

PERMANOVA confirmed significant differences in gene expression between groups (CTL, DAOM, C2) (Df = 2, R2 = 0.20277, F= 5.2141, p=0.005).

Supplementary Figure S8. Volcano plot of differentially transcribed maize genes

(A) Volcano plot of differentially expressed transcribed maize genes in the rhizosphere in DAOM197198 treatments vs controls and (B) in C2 treatments versus controls.

Supplementary Figure S9. Associations between fungal taxa and pht6

Positive associations between fungal taxa to pht6 expression in maize roots after AMF had been filtered from the dataset as they were the fungal taxa with the highest positive association with pht6 expression. No negative associations between fungal taxa and pht6 expression were found.

**References**

1. Oksanen J et al. vegan: Community Ecology Package. 2022. 2022. , 2.6-6.1

2. Love MI, Huber W, Anders S. Moderated estimation of fold change and dispersion for RNA-seq data with DESeq2. *Genome Biol* 2014;**15**:550. https://doi.org/10.1186/s13059-014-0550-8

3. Liu F et al. Systematic Identification, Evolution and Expression Analysis of the Zea mays PHT1 Gene Family Reveals Several New Members Involved in Root Colonization by Arbuscular Mycorrhizal Fungi. *Int J Mol Sci* 2016;**17**:930. https://doi.org/10.3390/ijms17060930

4. Willmann M et al. Mycorrhizal phosphate uptake pathway in maize: vital for growth and cob development on nutrient poor agricultural and greenhouse soils. *Front Plant Sci* 2013;**4**. https://doi.org/10.3389/fpls.2013.00533

5. Baker ME, Kahle D, King RS. TITAN2: Threshold Indicator Taxa Analysis. 2023. 2023.

6. Baker ME, King RS. A new method for detecting and interpreting biodiversity and ecological community thresholds: *Threshold Indicator Taxa ANalysis (TITAN)*. *Methods Ecol Evol* 2010;**1**:25–37. https://doi.org/10.1111/j.2041-210X.2009.00007.x

7. Martinez Arbizu, P. pairwiseAdonis: Pairwise multilevel comparison using adonis. 2020, R package version 0.4

8. Ihrmark K et al. New primers to amplify the fungal ITS2 region - evaluation by 454-sequencing of artificial and natural communities. *FEMS Microbiol Ecol* 2012;**82**:666–677. https://doi.org/10.1111/j.1574-6941.2012.01437.x

9. White TJ et al. Amplification and direct sequencing of fungal ribosomal rna genes for phylogenetics. PCR Protocols. Elsevier, 1990, 315–322.

10. Nossa CW. Design of 16S rRNA gene primers for 454 pyrosequencing of the human foregut microbiome. *World J Gastroenterol* 2010;**16**:4135. https://doi.org/10.3748/wjg.v16.i33.4135
